# Supplementary material for: Assessing the interplay between human mobility and mosquito borne diseases in urban environments
Source: Sci Rep. 2019 Nov 15;9:16911. doi: 10.1038/s41598-019-53127-z (PMC6858332; doi:10.1038/s41598-019-53127-z)
Supplement: Supplementary file 1 — Supplementary materials [file 41598_2019_53127_MOESM1_ESM.pdf]

Supplementary materials

Assessing the interplay between human mobility and mosquito borne diseases in urban environments

Emanuele Massaro*, Daniel Kondor, Carlo Ratti
*emanuele.massaro@epfl.ch

Mobility Models

We report the comparison between the four mobility models used in this research: i) derived from mobile phone data, ii) random, iii) derived from a Levy flight distribution and iv) derived from the radiation model. In Figure S1 we report the Pearson correlation (P_c) coefficient between the 3 models. In the scatterplots each point correspond to the flow (i.e. total number of commuters) from a location w to a location h. mobility models.

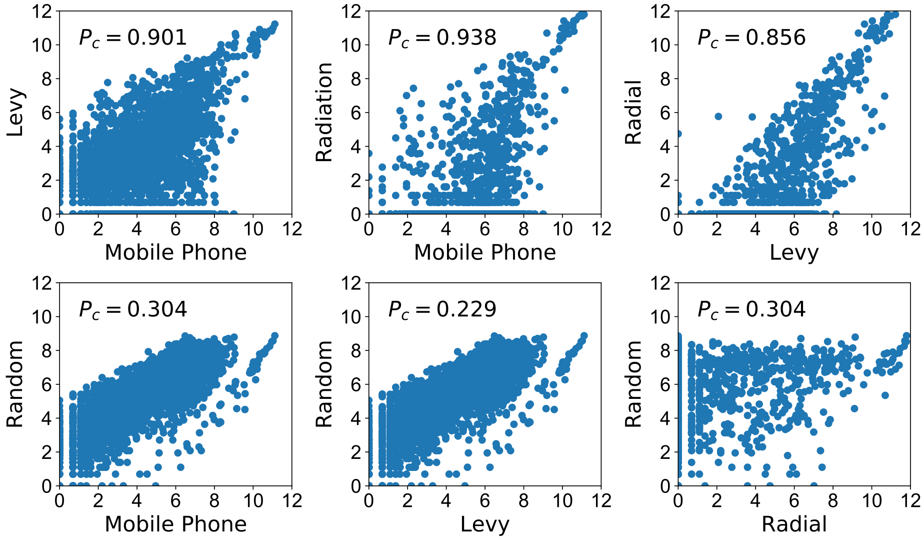

Figure S1. Correlation of amount of people travelling between the census areas: each point corresponds to the flow between two census areas.

Figure S2, Figure S3, Figure S4 and Figure S5 show displacement of the agents in their home and work locations for the different mobility models.

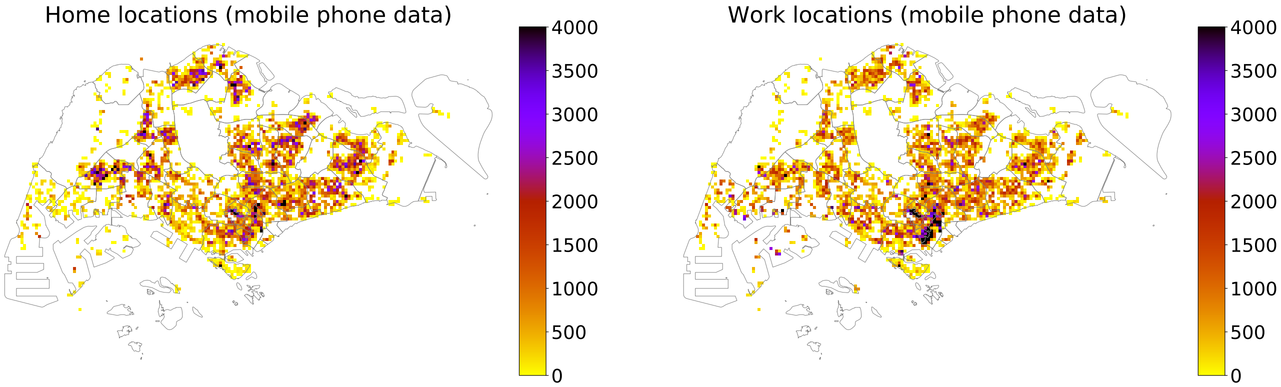

Figure S2. Home and work locations from mobile phone data. Count of users in the home and work locations respectively determined from the mobile phone dataset in each cell. The majority of jobs are located in the Central Business District, whereas the home locations are more equally distributed.

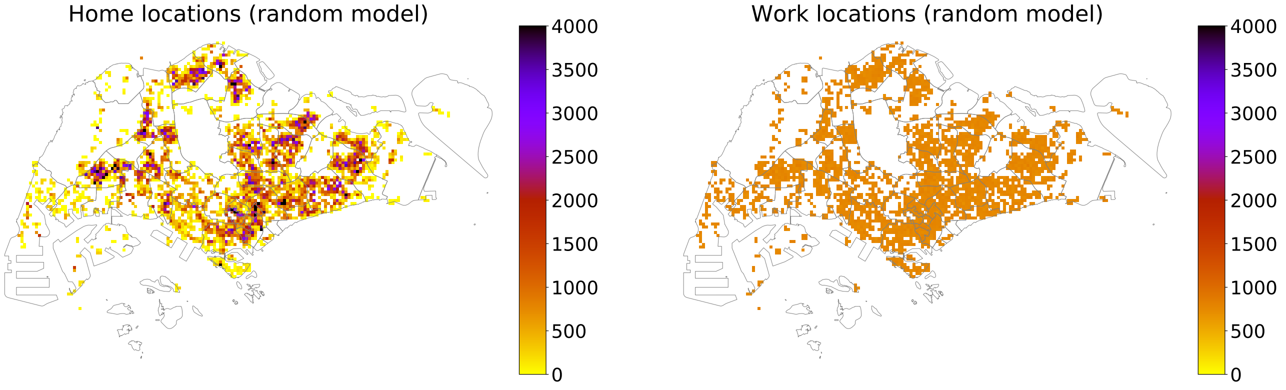

Figure S3. Home and work locations from the random model. The home locations are taken from the mobile phone data model while the work locations are randomly assigned.

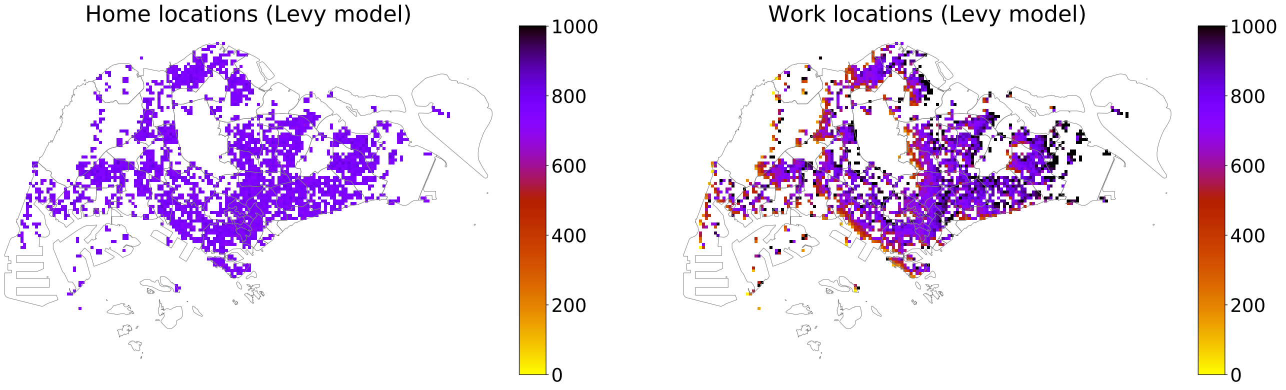

Figure S4. Home and work locations from the Levy model. The home locations are randomly assigned while the work locations are given with a distance from a Levy flight distribution.

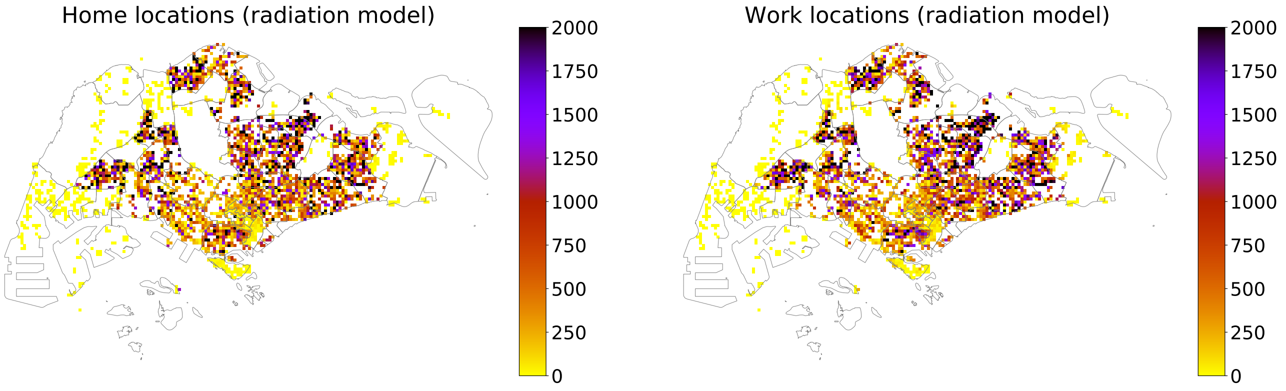

Figure S5. Home and work locations from the radiation model.. The home locations are assigned from the census data while the work locations are assigned with a distance following the radiation model.

Temperature dependent parameters

Most of the parameters (as reported in Table 1 in the main text) used in our methodological approach depend on the temperature. The equations governing those parameters are the following:

$$
\begin{aligned}
\varepsilon_A^v(T) &= 0.131 - 0.05723T + 0.01164T^2 - 0.001341T^3 + \\
&\quad +0.00008723T^4 - 3.017 \cdot 10^{-6}T^5 + 5.153 \cdot 10^{-8}T^6 - 3.42 \cdot 10^{-10}T^7 \\
\mu_A^v(T) &= 2.13 - 0.3787T + 0.02457T^2 - 6.778 \cdot 10^{-4}T^3 + 6.794 \cdot 10^{-6}T^4 \\
\mu_V^v(T) &= RHF * (0.8692 - 0.1599T + 0.01116T^2 - 3.408 \cdot 10^{-4})T^3 + 3.809 \cdot 10^{-6}T^4) \\
\theta_A^v(T) &= -5.4 + 1.8T - 0.2124T^2 + 0.01015T^3 - 1.515 \cdot 10^{-4}T^4 \\
\gamma_V^v(T) &= \frac{(3.3589 \cdot 10^{-3} * Tk)/298exp((1500/R)(1/298 - 1/Tk))}{1 + exp((6.203 \cdot 10^{21})/R * (1/(-2.176 \cdot 10^{30})) - 1/Tk)}
\end{aligned}
$$

where Tk is the degrees in kelvin.

$$
\begin{aligned}
\phi^{h \to v}(T) &= 1.004 \cdot 10^{-3}T(T - 12.286) \cdot (32.461 - T)^{1/2} \\
\phi^{v \to h}(T) &= 0.0729T - 0.97.
\end{aligned}
$$

We have also included an adult mortality factor based on relative humidity[1]. Temperature and relative humidity are converted to a vapor pressure measure, $VP = 6.11 \cdot 10^{(7.5T/273.3+T)/10}$. This value is converted to a relative humidity factor RHF) based on the following rules: If $10 < VP < 30$, $RHF = 1.2 - 0.2 \cdot VP$, and if $VP \geq 30$, $RHF = 0.5$.

In Figure S6 we show the values of the described parameters for temperature between -10°C and 40°C while in Figure S7 we show the values of the parameters during the years 2013 and 2014.

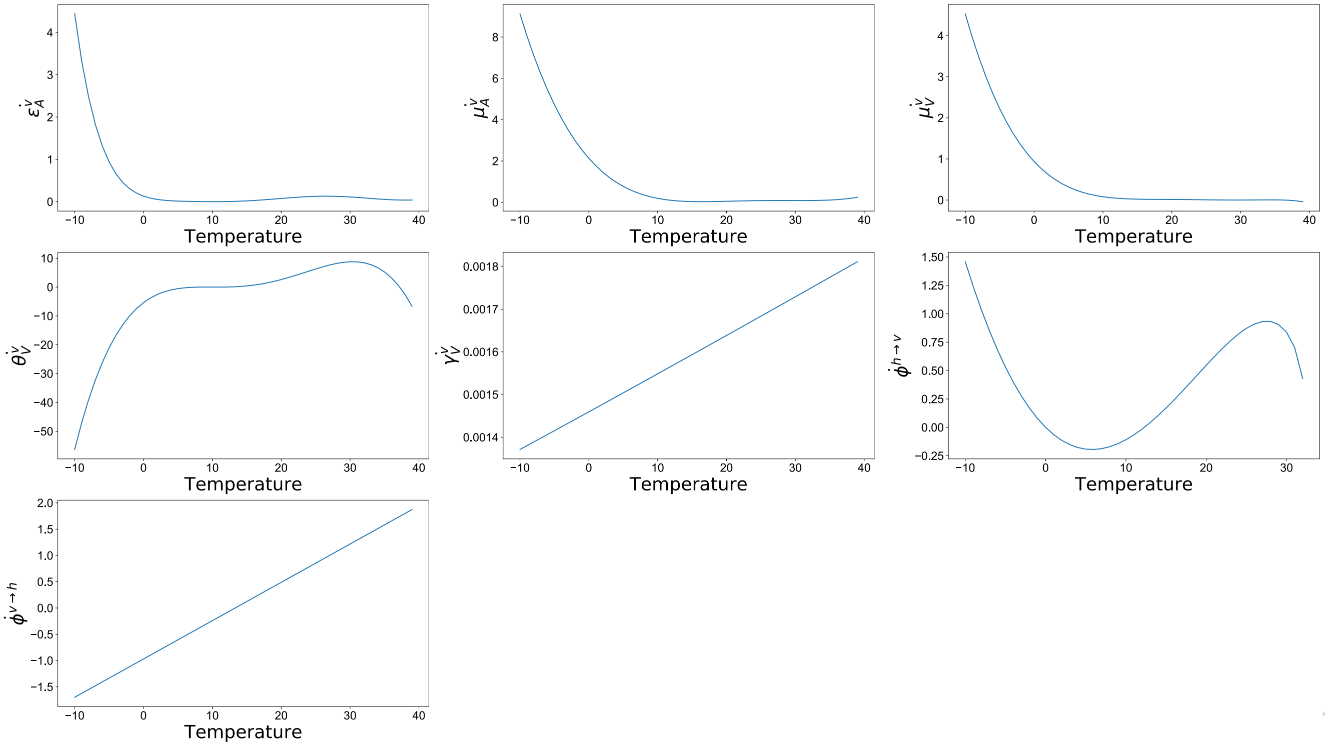

Figure S6. The temperature-dependent parameters as function of the temperature.

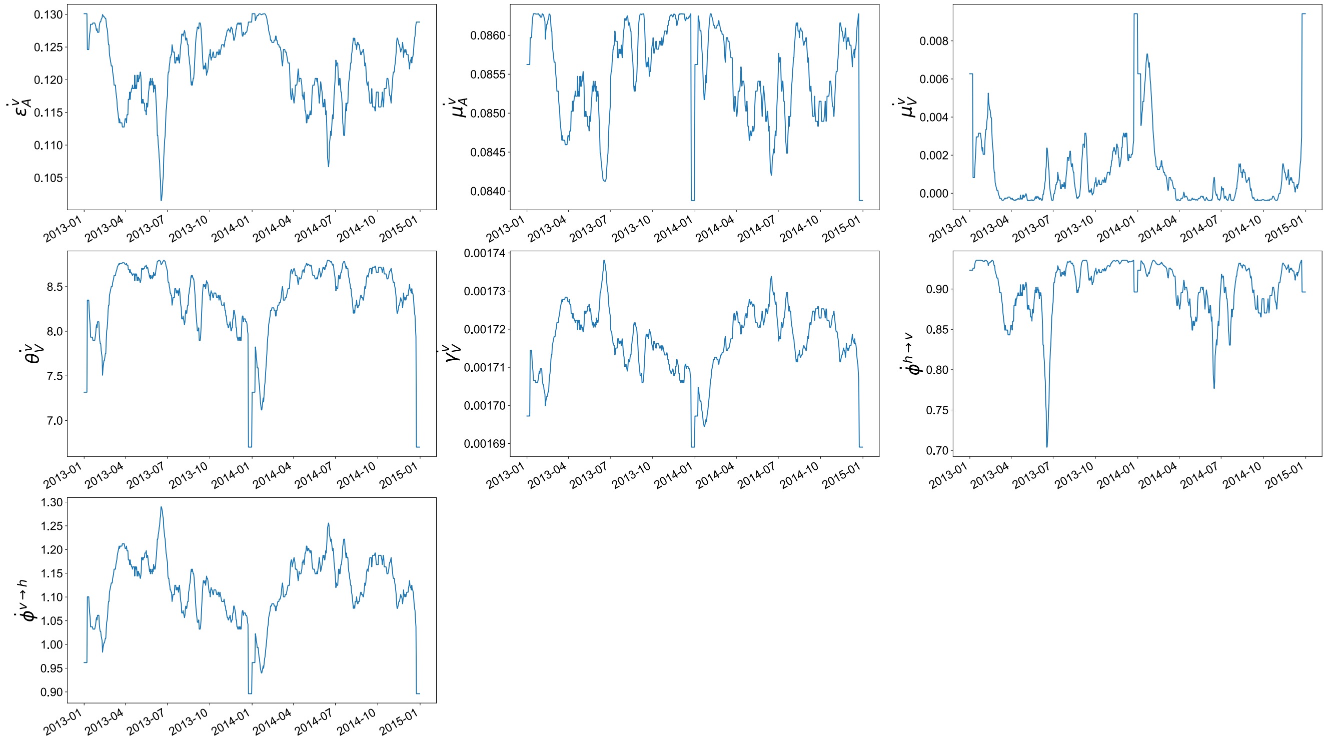

Figure S7. The temperature-dependent parameters used in the ento-epidemiological framework for Singapore in 2013-2014.

Structural Similarity Index

In order to compare and quantify the spatial prediction of the simulations with the real case scenario, we use the *structural similarity index*. The Structural SIMilarity (SSIM) index is a method originally proposed for measuring the similarity between two images, but is applicable when comparing structural properties of 2-dimensional data, i.e. the spatial distribution of dengue cases in our case. The SSIM index can be viewed as a quality measure of one of the images being compared, provided the other image is regarded as of perfect quality. It is an improved version of the universal image quality index proposed before[2,3] and is computed as:

$$SSIM(x,y) = \frac{(2\mu_x\mu_y + c_1)(2\sigma_{xy} + c_2)}{(\mu_x^2\mu_y^2 + c_1)(\sigma_x^2 + \sigma_y^2 + c_2)} \tag{1}$$

where x and y are appropriate-sized windows of the images to compare, where μ_x and μ_y are the average of x and y, σ_x^2 and σ_y^2 are the variances of x and y while σ_{xy} is the covariance of x and y. The parameters $c_1 = (k_1L)^2, c_2 = (k_2L)^2$ are two variables to stabilize the division with a weak denominator, where L is the dynamic range of the discrete pixel values. The two additional parameters are $k_1 = 0.01$ and $k_2 = 0.03$ by default. To obtain a similarity metric between two images, the SSIM values are averaged over all possible subsections of the images, defined by sliding windows of size 7×7 pixels.

The range of the value of the SSIM index is between 0 and 1: when two images are nearly identical, their SSIM is close to 1. For each epidemiological week in the period we compute the SSIM between the real case and the three simulated scenarios using the the `Python` function `structural_similarity` from the package `skimage`[1]. An example of SSIM in a toy grid is reported in Figure S8.

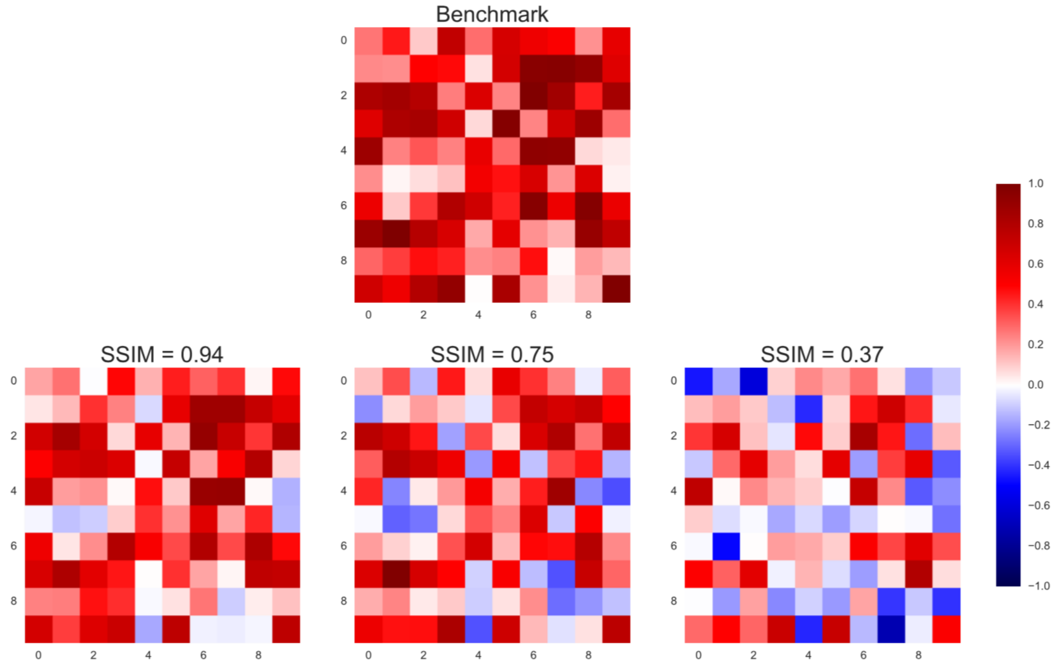

Figure S8. Structural similarity index (SSIM) illustration. We generated a 10×10 grid in which in each cell we assign a random number between -1 and 1. This grid is mathematically described by a matrix $B^{10\times 10}$ in which each element of the matrix $B_{i,j}$ is a random number between -1 and 1. This matrix represents our benchmark to test the SSIM index. We then generate other three grids (from left to right bottom) starting from the benchmark in which for each $B_{i,j}$ we add or subtract random number between 0 and 1 times 0.25, 0.5 and 0.75 respectively. In this way we are able to compare three different scenarios with the benchmark with different degree of difference from the original one. As we can observe similar images generate greater SSIM if compared with the benchmark.

[1] http://scikit-image.org/docs/dev/auto_examples/transform/plot_ssim.html

References

1. Wesolowski, A. *et al.* Impact of human mobility on the emergence of dengue epidemics in Pakistan. *Proc. Natl. Acad. Sci.* (2015).

2. Wang, Z., Bovik, A. C., Sheikh, H. R. & Simoncelli, E. P. Image Quality Assessment: From Error Visibility to Structural Similarity. *IEEE TRANSACTIONS ON IMAGE PROCESSING* **13**, 600–612, DOI: 10.1109/TIP.2003.819861 (2004).

3. Wang, Z. & Bovik, A. C. Mean squared error: love it or leave it? a new look at signal fidelity measures. *IEEE signal processing magazine* **26**, 98–117 (2009).

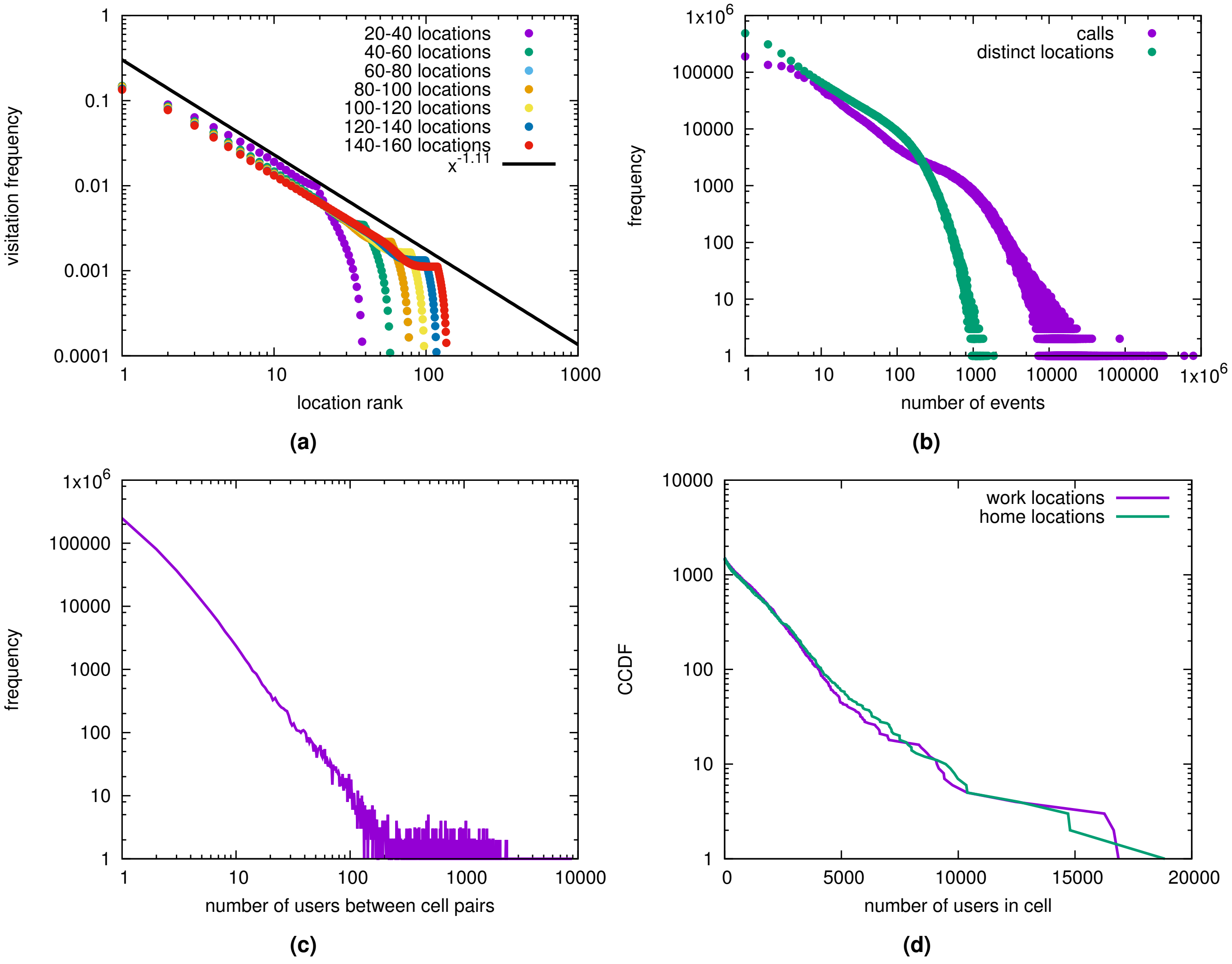

Figure S9. Statistical analysis of mobile phone data. A) Distribution of number of locations (antennas). B) events (calls / texts) per user and distribution of visitation frequencies of user locations as a function of location ranks (here the locations still refer to antennas). C) Distribution of number of locations (antennas) and (b) events (calls / texts) per user and distribution of visitation frequencies of user locations as a function of location ranks (here the locations still refer to antennas). D) Distribution of the commute matrix elements (i.e. the number of users who commute between any two cells).

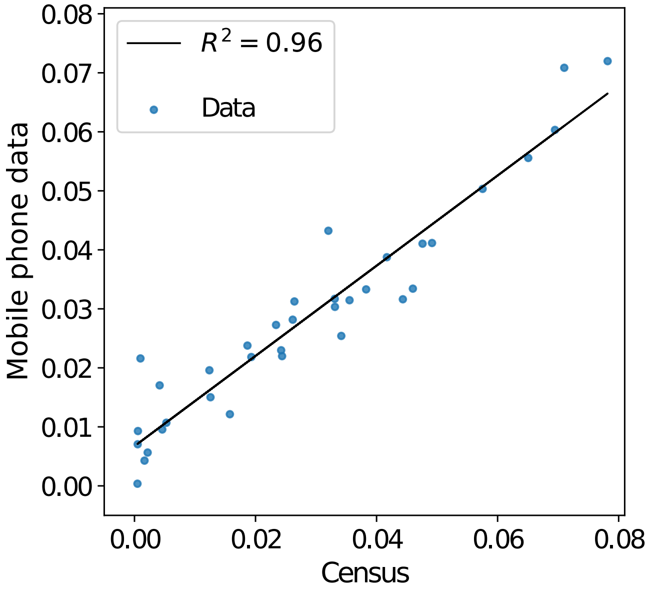

Figure S10. **Fraction of the population in Singapore's districts** according to the 2010 census versus the home locations determined from the mobile phone dataset. With a correlation coefficient of 0.96, the two spatial distributions are highly linearly correlated.

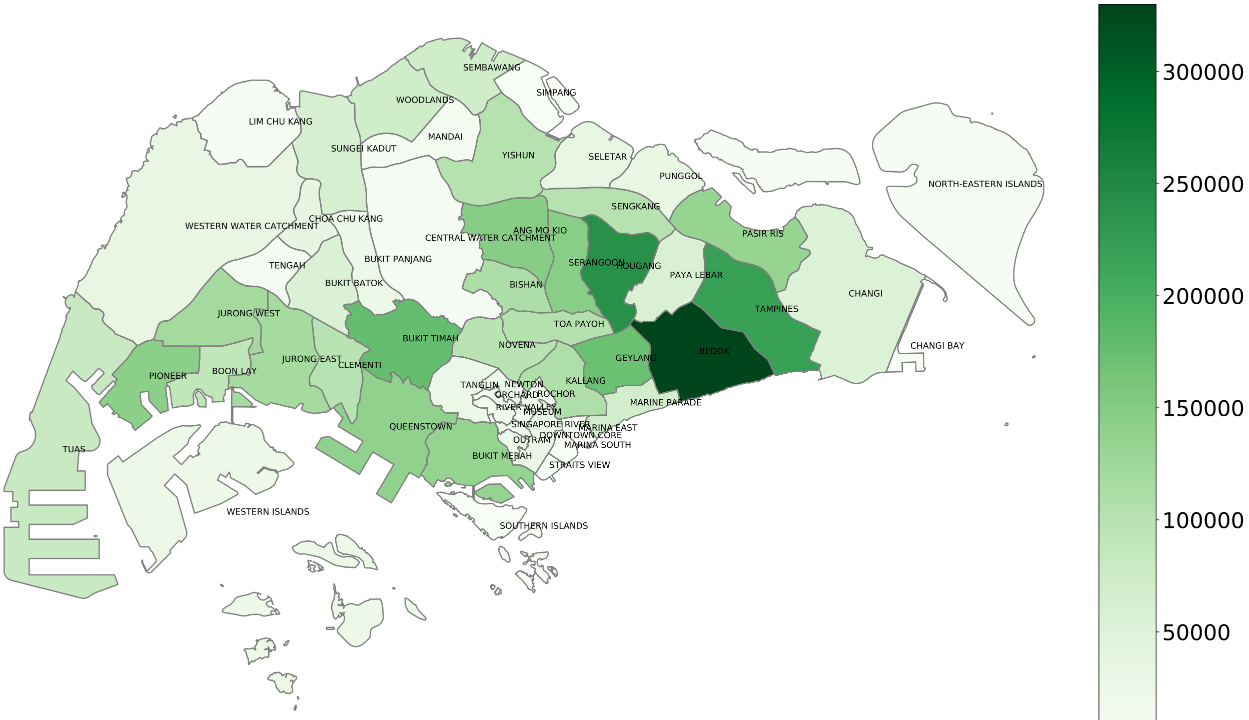

Figure S11. Basic demographic characteristics of the Singapore resident population by their registered place of address from the Census of Population 2010. The Singapore resident population comprises Singapore citizens and permanent residents. Of the 3.77 million Singapore residents as at end-June 2010, about 57% were concentrated in ten planning areas. There were five planning areas with more than 200,000 Singapore residents. Bedok, Jurong West and Tampines each had more than 250,000 Singapore residents, with Bedok having the most number at 294,500 in 2010. The other two planning areas with more than 200,000 Singapore residents in 2010 were Woodlands (245,100) and Hougang (216,700). Shapefile data are downloaded from the Singapore open data portal https://data.gov.sg, while population data are downloaded from https://www.worldpop.org/

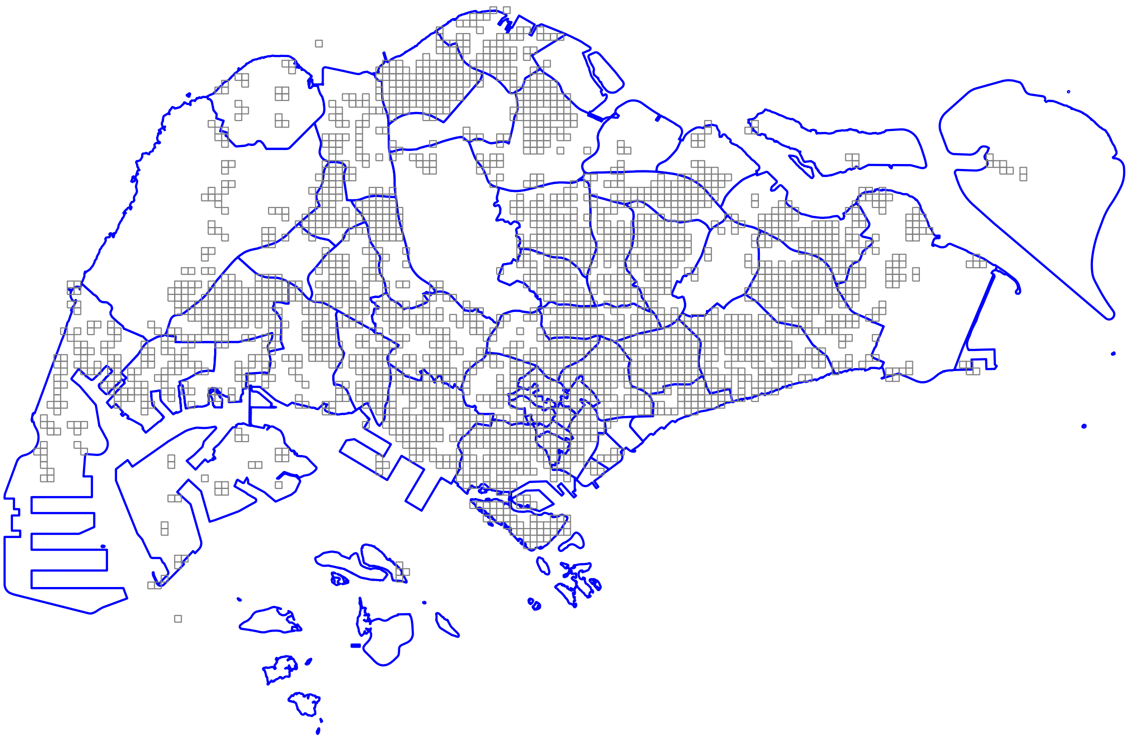

Figure S12. The 2598 cells used in this research.

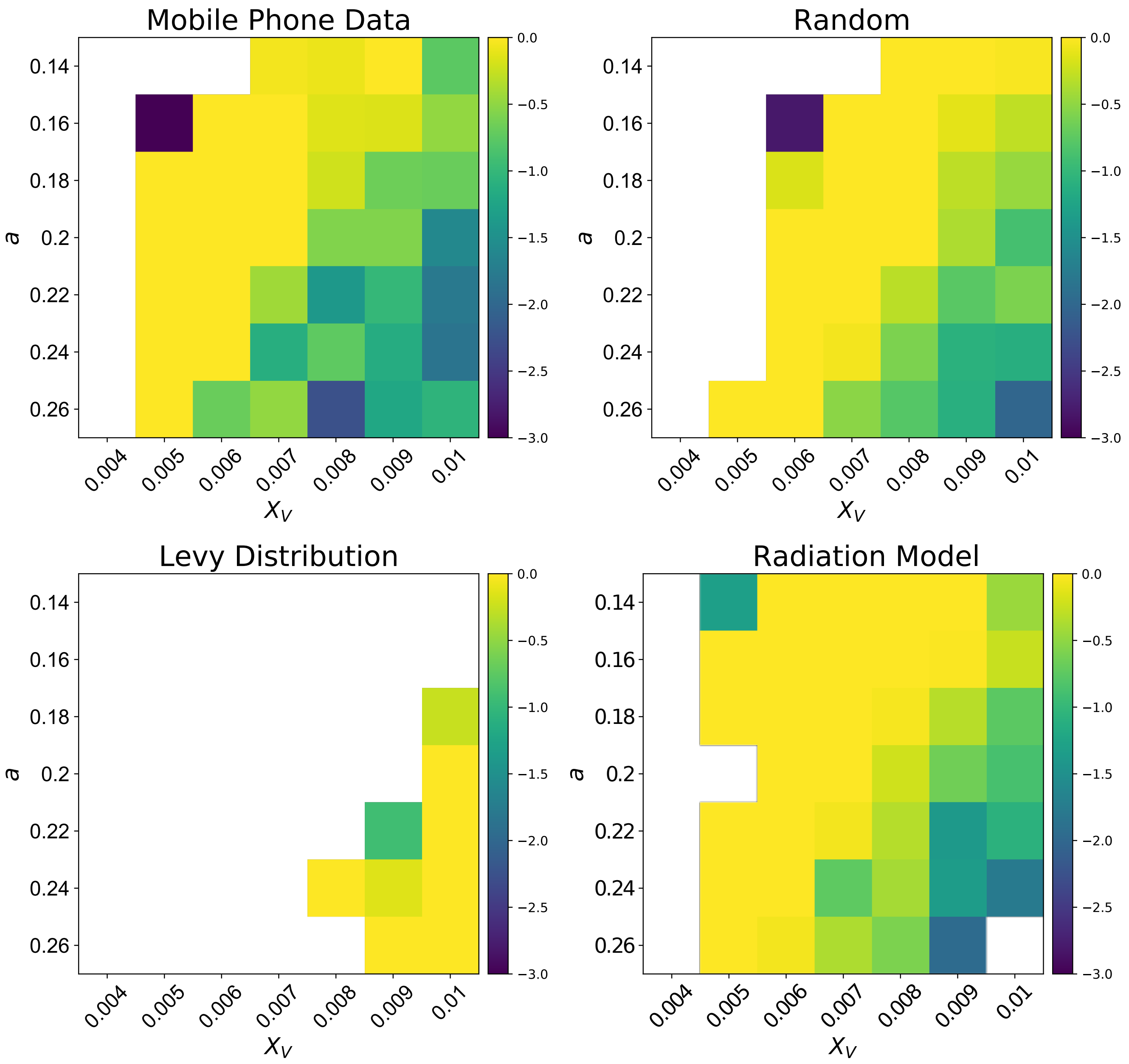

Figure S13. Logarithmic values of the R^2 of the temporal estimation for each couple of parameters in the different mobility models where the white spaces correspond to the negative value of the R^2.
